# The blind spot of online creative idea generation studies: A perspective of media materiality

**DOI:** 10.3389/fpsyg.2022.976193

**Published:** 2022-11-03

**Authors:** Siyu Liu, Feng Ji, Cen Zeng

**Affiliations:** ^1^Faculty of Humanities and Arts, Macau University of Science and Technology, Taipa, Macao SAR, China; ^2^School of Public Administration, Hohai University, Nanjing, China; ^3^School of Journalism and Communication, Guangzhou University, Guangzhou, China

**Keywords:** online creative idea generation, digital technology, media materiality, material metaphor, mass psychology, creativity

## Abstract

Online creative idea generation is often considered an extension of traditional creative idea activities on the Internet platform, in which digital technology plays an important role. Consistent with the studies on traditional creative idea activities, the studies on online creative idea generation take the creativity of mass psychology as the core, and believe that digital technology can stimulate people’s creative output. This study challenges the past research paradigm from the perspective of media materiality, redefines the processes and activities of online creative idea generation, and further suggests that it may affect people’s psychology by reducing their creativity without real awareness. The study is innovative in both theory and practice. We also discuss the limitations and future directions of the study.

## Introduction

Online creative idea generation is the practical activity of developing unique ideas for a specific issue or task through the Internet or digital technology. It is generally believed that traditional creative generation activities are behaviors such as the design that people perform in the real world (Goel, 2014). Therefore, the psychological factors associated with individual creativity have become the focus of scholarly discussion. For example, one study established the influence of positive and negative moods on creativity production (Friedman et al., 2007). Using the experimental approach, they examined the relationship between moods and creative generation tasks. In addition, Ward et al. (2004) explored the potential role of specificity and abstraction in creative idea generation activities. In the Internet era, the application and development of digital technology have shifted people’s creative production from offline to online and from the real world to virtual space. This means that online creative idea generation is the digital extension of traditional creative generation activities and is the product of combining creative generation activities with digital technology.

The relationship between technology and human creativity has become one of the key concerns in the field of online creative idea generation. Scholars have discussed how digital technologies have distinguished between online and offline creative generation activities, the online environment, and user characteristics of online creative generation activities. However, all these studies are based on anthropocentrism. In recent years, due to the flourishing of post-humanism and new materialism, media materiality has provided a unique emerging perspective for our study. Post-humanism argues that modern technology is so integrated into people’s lives that humans no longer seem to be purely natural but rather technologically artificial (Porpora, 2017). In this sense, post-humanism challenges the biases in mainstream sociology and media studies (Gane, 2005). Similarly, new materialism is a theory that has been very influential in the West in the last decade or so, with a variety of theoretical schools shifting their interest and focus toward the discussion of the material. New materialism advocates a shift away from the human as the center of research, and instead, academic research is conducted through the view of the material, emphasizing the interaction that occurs between the material and the human (Tuin and Dolphijn, 2012; Gamble et al., 2019). Obviously, the perspective of media materiality is chronically the blind spot of online creative idea generation studies.

Based on these, this conceptually-driven study tries to explore the online creative idea generation process supported by digital technologies from the perspective of media materiality. This study attempts to challenge the previous research paradigm of online creative idea generation, refusing to regard it simply as a tool used by people, but as a form of media with the activity. We advocate that both humans and technologies should be regarded as actors in the practice of online creative idea generation, which will help us to have a deep understanding and redefine this unique creative idea activity. More importantly, our study which is based on media materiality will also discuss the relationship between online creative idea generation and mass psychology, especially people’s creativity. As digital technologies have become a part of creative idea generation activities, they have almost completely changed traditional creative idea generation and become a new way of creativity. It subtly affects people’s cognitive psychology through material metaphor, which may impair people’s creativity as never before, but we are not aware of it.

## Literature review

### From humans to materials: The materiality turn of media studies

Exploring online creative idea generation from the perspective of materiality is a great challenge. It may incur skepticism from some scholars. Where is the material? What is materiality? How is materiality manifested? Indeed, in previous social science studies on technology, such as the Internet, it has been considered immaterial virtual cyberspace and, thus, can be viewed in isolation from the material world (Hondros, 2015). However, Blanchette (2011) argues that it is problematic to discuss online information in the digital era solely as immaterial and that although information on the Internet appears immaterial, it cannot exist apart from its material form. Digital technologies are increasingly changing people’s cognitive psychology and behavior, but the discussion of their materiality has rarely been addressed by academics. Thus, Murdock (2018) critically notes that materiality has long been a blind spot in communication research. In approximately 2010, scholars began to shift their perspectives on the Internet, and studies of media materiality began to emerge (Bennete and Joyce, 2010). That is, a “material turn” in Internet studies is now taking place (Parikka, 2012; Herzogenrath, 2015).

The material turn in media studies is not accidental but has diverse theoretical origins. For Mukerji (2015), Marxism, Foucault’s thoughts on panopticon and surveillance, and science and technology studies (STS) are the most important theoretical foundations of materiality studies. In addition, new materialism and Heidegger-inspired object-oriented ontology have also driven the emergence and development of materiality studies (Zhang and Zhang, 2019). The current studies of the materiality of digital technologies are based on two main dimensions. One of them is the subject-object dimension, and the other is the real-virtual dimension. In the subject-object dimension, researchers usually consider traditional analysis to be an anthropocentric way that treats humans as subjects and materials as objects. In this way, humans are active and materials are passive, with a subject-object relationship between them. The anthropological research tradition rejects the material as an object, arguing that the material should likewise be seen as a subject and possess activity (Rowlands, 2005). This train of thought criticizes anthropocentrism and emphasizes that the relationship between humans and materials should be an inter-subjectivity. The dimension of real-virtual reflects the fact that the material in our studies includes concrete material entities and whether it should also include materials that are not entitative, such as software, algorithms, and application program (APP). The German media theorist Kittler, who makes the classic statement “there is no software,” overcomes the shortcomings of classical hermeneutics by considering computers, computational structures, and their programs as materials (Kittler, 1992). In his view, underneath the software is the hardware, and underneath the hardware, there are substances such as water and electricity that produce the hardware as infrastructure (Kittler, 1992).

How could the materiality of media technology be understood? Two Chinese scholars use a step-by-step dismantling method, specifically in the vein of “thing-material-materiality (as a noun)-materiality (as an adjective),” and briefly analyze the smartphone as an example (Zhang and Zhang, 2019). To be more specific, the smartphone is a “thing,” and the metal, glass, silicon, aluminum, copper, etc., that make it up are “material,” and the properties that distinguish it from humans are “materiality (as a noun),” while a series of practices associated with it is “materiality (as an adjective).” In other words, things are not only materially constituted but also may be represented by further materials (Fuchsberger et al., 2013). The studies of materiality introduce the method of material object analysis and distinguish mainly between object-centered and object-driven approaches. The former approach focuses on the studies of things in themselves, i.e., the discovery of their nature in a depictive way. The latter approach, on the other hand, considers materials not as passive but as active. Materials not only reflect the values of their time but can also create meaning in themselves and thus cannot simply be imagined as things to be used by people but aim to understand the relationship between the making and use of materials and people, nations, and cultures. Material object analysis generally examines three aspects related to a particular media material, i.e., affordance, restriction, and coding process. To date, several scholars have used this method to study media materials in the Internet era. For example, one study specifically explores the meaning-making and functional use of computer icons as signs (van den Boomen, 2008). Gerlitz and Helmond (2013) study Facebook’s Like buttons and find that Like buttons enable the flow of multiple data between different users, contributing to the simultaneous decentralization and recentralization of the Internet. By exploring the creation and technical infrastructure of Like buttons, researchers prefer to think of them as a “Like economy” rather than the more social web experience that Facebook itself claims to be. Allen-Robertson (2017) enriches our understanding of digital media by examining four digital media, such as the hard disk drive, phonograph, magnetic tape, and optical media, showing that the material reality of contemporary digital technology is still present and continues to influence our minds and actions. All of these studies call for us to value the various media materials in the Internet environment. They may or may not be visible, but they are all material of the digital era and have materiality.

### The theoretical background of media materiality

Regarding media materiality, it must first involve how to treat technologies. Latour, the prominent philosopher of technology, proposes that the key to understanding technology lies not in resolving the debate between technological determinism and social determinism, but in the ontology of subject and object between humans and technology and that the only way to resolve this is to find new explanations that can replace the dichotomy (Khong, 2003). Latour absorbs the theoretical ideas of semiotics, accepts Whitehead’s concept of the actual entity, develops Callon’s thoughts, and then proposes the expression actant, actor, or agent. Latour’s purpose of using actor, agent, or actant is that they can be anything, individual or collective, human or non-human (Latour, 1993). All actors exist equally but at the same time are not equal. They are equal in that no actor can be constructed by other actors, and they are not equal in that each actor contributes to the assembling in different degrees (Bryant, 2011). Latour’s main aim in invoking networks is to incorporate human and non-human actors in the same capacity, where each actor is a node of the network, equal and decentralized, the only difference being the number of connections to other nodes, but equal in importance and network status (Latour, 1999). Thus, the essence of the actor exists in the connection with the other. It can be said that there is no actor without a network, and there is no network without an actor. The actor-network is a breakthrough from the ontology of nature and society, object and subject in the philosophy of technology (Latour, 1996). The representation of technical artifacts in terms of the networked actor makes them no longer things that exist dependent on subjects, but the result of various chains of relations, the fundamental properties of assembling. Theoretically, the distinction between humans and things is no longer the most important, but their representation and the social effects they construct are more crucial (Waltz, 2006). As can be seen, Latour’s notion of giving technical artifacts to actors goes beyond the previous traditional ideas of objects and tools regarding technology. Latour redefines technology in terms of actors, providing the basis for media materiality.

Kittler focuses on the materiality of media technologies. He deliberately avoids the active role of humans in media technology, treating the human as a mere object or referent of media analysis and internalizing the other in the structure of media technology, dissolving the anthropocentric status of activity (Guo and Zhao, 2021). In his monograph *Gramophone, Film, and Typewriter*, Kittler (1999, p. 1) states bluntly that “media determine our situation.” While this statement may seem to be distinctly media determinism, the German media theory represented by Kittler does not adhere to purely technological determinism, nor does it preach a purely social determinism. This theoretical perspective holds that technology is determined by a specific material and its conditions and is closely related to the social environment, which in turn is influenced by the material. It moves completely away from the dichotomy of two kinds of determinism and instead takes a pioneering stance in discussing the relationship between media technology, society, and humans. For Kittler, the media is a transcendental existence, and the concept of the human is both born or derived from the media. Meanwhile, the media technology simultaneously annihilates the concept of the human. In this way, the media machine reshapes people’s perceptions and consciousness, and human activity is completely obscured by the media. Kittler emphasizes media materiality to advocate the idea of an intelligent machine with automated learning and evolutionary capabilities as a subject that can cosmfortably control and process information rather than a tool for human manipulation (Guo and Zhao, 2021).

The maverick scholar Flusser creates a body of communication theory called Communicology, which has attracted increasing international attention in recent years. One of the most distinctive aspects of his communication thought is his focus on the nature of communication, with the media as the basis of social reality. He classifies the information or content of human communication into three types: epistemological information of knowledge, ethical information of desire, and aesthetic information of sensation/emotion (Flusser, 1990). Knowledge information is non-sense if it does not have ethical and aesthetic characteristics. Thus, Flusser argues that computers only have the ability to be knowledgeable. From this perspective, it seems that artificial intelligence technology in the digital era is incapable of ethical and aesthetic judgment and possessing knowledge information. Flusser thinks that in communication activities, various ideological discourses must package themselves as de-ideological, masquerading as scientific knowledge (Finger et al., 2011; Poltronieri, 2014). He views the way technology operates as such an ideology, emphasizing that the way technology operates hides people’s behavioral patterns and that the way people operate becomes a technological apparatus that is ostensibly ideologically uninvolved. In *Toward a Philosophy of Photography*, Flusser introduces the concept of apparatus. An apparatus is a product made by man using scientific or mathematical theories to create, process, and store symbols (Flusser, 2013). In Flusser’s opinion, the physical device or program is less important than the theory that determines its design. The apparatus program is a system of combinations of possible elements, and the power belongs to the person who designed the program (Flusser, 2013). The intellectual origin of this idea of Flusser is Descartes. Descartes emphasizes that a rational way of thinking should break down problems into their basic elements. Using photography as a material metaphor, Flusser (2013) prefers that the image produced by a camera is not an image taken by a person in the traditional sense but rather an image produced by a machine. In fact, people have long accessed the ability to represent the world they see in a pictorial way into images. That is, to turn a four-dimensional world and a three-dimensional experience into a two-dimensional plane. At the same time, people have the ability to imagine the four-dimensional world by looking at the two-dimensional plane (Flusser, 2011). Flusser (2013) reminds us that photography represents the program, not the real world.

Through a brief literature review, the theoretical significance of studying online creative idea generation from the perspective of materiality has become clear. As digital and artificial intelligence technologies continue to penetrate people’s daily lives, the original atypical materials are also included in the category of media, and the original media derive new material forms (Zhang and Zhang, 2019). Online creative idea generation is a typical representative of them. The perspective of media materiality provides us with abundant academic imagination.

## Analysis

### The activity of technology in online creative idea generation

Previous studies have typically treated humans as creative subjects (e.g., Shen et al., 2016; Steils and Hanine, 2019). In this concept, online creative idea generation technology is nothing more than a clever combination of creative activity and digital technology. It is software that is used by people, thus, it is humans who have the activity of creativity. However, the perspective of media materiality provides us with a new way of interpreting online creative idea generation.

First, according to Latour, we must consider online creative idea generation technology as the actor. The traditional thought of dichotomy makes people always subconsciously view things as objects of the subject when they describe things. Latour reminds us that we should return to the essence of technical artifacts to find the way out of the dichotomy. In Latour’s opinion, all actors are active and have action. Since actors are in action all the time, the whole network is dynamic, and assessing the status and role of actors requires a reinterpretation of what an actor is each time (Latour, 2011). Latour (1999) gives an example of a murder with a gun. When a person with a gun kills another person, is it a “gun killing a person” or “a person killing another person?” Latour argues that when a person has a gun in his/her hand and uses it to kill, the person becomes the “murderer,” the gun becomes the “murder weapon,” and the victim is transformed from a person to a “corpse.” This process is the “translation” between actors. The act of “killing” is neither the result of the shooter’s intention nor the result of the gun’s firing, but the result of an actor-network formed by the interaction between the person and the gun. Hence, both the person and the gun are actors in the process. Along this argument, when people are using online creative idea generation programs for creative activities, is it the people who are doing the idea generation or the digital technology? Obviously, in the activity, the person becomes the designer, the program becomes the design platform, and the designed product becomes the final result. The translation is produced among the three, and the online creative idea generation activity becomes the result of the action network formed by the interaction between the person and the program. Therefore, from this perspective, online creative idea generation should not only be regarded as a tool but also as an actor.

Second, technology necessarily has a material element (Ihde, 1979). In the Internet era, “[h]uman-machine relations are existential relations in which our fate and destiny are implicated, but which are subject to the very ambiguity found in all existential relations” (Ihde, 1979, p. 4). The machine becomes the environment of human existence; it is everywhere and becomes a part of our being. An online creative idea generation program is just such an intelligent machine. In Kittler’s core idea, the media not only influences people’s perceptions but also even opens up our existence. The media makes “what is” possible. “Sense perceptions had to be fabricated first” (Kittler, 1999, p. 3), and this happens through the media. It is obvious that online creative idea generation technology opens up the existence of online creative activities which makes the generation program become the creative media. Because of this, humans can select, produce, and store data to generate ideas online. In other words, as a media, the online creative idea generation process has its own set of logic and does not operate by human logic. As Kittler points out, with the advent of contemporary digital technology, our information and data become encoded using different programs that then present specific interfaces. Similarly, the interpretation of apparatus also implies that although people operate the program technology, it is actually the program technology that disciplines us (Flusser, 2013). Online creative idea generation programs are infinitely reproducible as the media that can produce images, artistic texts, or logos. This reproducibility means various selections and combinations of programs making designers become the extension of online creative idea-generation programs. That is, each product produced by online creative idea generation corresponds to a specific combination of elements in the program. It also reflects the activity of technology in online creative idea generation.

In sum, from the perspective of media materiality, we should not simply regard online creative idea generation as a tool but should think about the co-existing relationship between people and technology in the process of online creative activities. Only in this way, the value and significance of humans in online creative generation can be highlighted. Thus, online creative production is a media form of mutual shaping of machines and humans, and the process of making creative production activities together. It is the process of interaction between humans and machines together as subjects.

### The influence of online creative idea generation on mass psychology associated with individual creativity

Kittler (1999) borrows a quote from Nietzsche, emphasizing that our collaborative tools are also involved in our psychology, to demonstrate that any media can influence the human’s consciousness patterns. Traditional research on creativity support systems has argued that these tools based on information technologies could increase the creative output of individuals or groups (Müller-Wienbergen et al., 2011; Althuizen and Wierenga, 2014; Wang and Nickerson, 2017). That is, when idea generation is supported by digital technologies, people’s creativity can be further enhanced. However, from the perspective of media materiality, digital technologies may restrict people’s creative output.

First, while creative designers benefit from technology affordances, they are bound to be constrained by them. Kittler considers that material technologies get power by disciplining us and the power is inherent in media technology. Online creative idea generation programs also own the technical power naturally. At the same time, Kittler (1999) inherits Lacan’s distinction between the symbolic, the imaginary, and the real, arguing that human psychology and our mental organs are increasingly perceived as information machines, and their eventual connection or merging becomes increasingly thinkable. Thus, online creative idea generation programs are such creative technological substances that change the state of people’s creative production and the results of their creative production. Online creative idea generation technology not only provides us with the convenience of idea production but also makes human creative activities subject to some restrictions. This is the result of the inter-embedding of the technology affordances with the restrictions, which can be considered the algorithm agency. After the invention of the computer, human society entered the digital era, and all symbolic systems were turned into numbers, which completely stripped people from the technical media, and this was the reality that people could not reach. The automation and autonomous computing ability of computers, as well as the current autonomous evolution and learning ability of artificial intelligence, are manifesting the existence of media subjectivity. Therefore, traditional creative idea generation requires creative professionals to develop rich imaginations, brainstorm, and apply innovative ideas to the artistic design of a product (Ebigbagha, 2020). The affordances of online creative idea generation technology seem to enable everyone to have such ability quickly while restricting the possibility of generating more ideas.

Second, inspired by Flusser’s Communicology, we can consider digital technology as a way to influence mass psychology and behavior through material metaphors. According to Flusser (2011), the value of humans has been greatly weakened by the rapid development of science and technology and has become the raw material for scientific manipulation and interpretation in the era of the Internet. It means that online creative idea generation based on digital technologies is completely different from traditional creative activities. Online creative idea generation products are produced according to the specific technical principle that is written as text. Therefore, online creative idea generation products are abstracts of texts. Furthermore, the texts are based on the abstracts of traditional products, which are abstracts of the real, so the online creative idea generation products are actually the third abstracts of the real. Flusser’s explanation of the abstract may be very difficult to understand, but we should still be clear about the core opinion, which is that online creative idea generation products have become completely different from traditional creative idea generation products.

Being in the world of apparatus means that we recognize, experience, and evaluate the world through apparatus (Flusser, 2013). However, apparatus keeps tricking us by trying to make us forget that computers are set programs. The process of online creative idea generation involves operators who must make choices based on the program’s possibility system. That is, online creative idea generation programs are systems of possibilities for people to choose from, and each idea production activity is a combination of these choices. Therefore, Flusser considers that in a computer, the freedom of any software package user is also programmed freedom (Amerika et al., 2010). More critically analyzed, this apparatus is a black box. People know how to use it, but most of them do not know how it works (Flusser, 2013). Hence, on a psychological level, especially for some less creative people, people want online creative idea generation programs to become increasingly simplified and easier to operate. For example, people want to be able to design a company logo with just a few keystrokes. Users usually think that there is no need to decode or understand how the program works. As a result, users become an extension of the online creative idea generation process, and their actions become automatic idea production. Ultimately, Flusser metaphorically represents the entire society as a giant black box (Zhang, 2020), including the financial industry, industrial development, and the educational system. We take these for granted and adapt to our role as users without making any challenges.

In a word, as communication media is based on digital technology, online creative idea generation is affecting people’s psychology and behavior. Because of the restriction of technology itself, the way it works tells us what to do and what not to do. People also accept it psychologically by default and translate it into their own behavior. Over time, people may become less creative. In addition, because online creative idea generation is active, people often cannot rely entirely on their own creativity for their online creative activities resulting in alienation between human and human-made materials (Schwendener, 2016). Thus, we should be alert to the dangers of this alienation. In the future, we can exercise our creativity as humans by challenging the restriction set by technology and developing critical thinking. This is the attitude one should maintain in response to the future of online creative idea generation.

## Conclusion and discussion

This study analyzes online creative idea generation from the perspective of media materiality, challenging the long-standing anthropocentric research paradigm and highlighting the importance of material in the Internet era. Theoretically, this study contributes to the literature on online creative idea generation by redefining this creative idea activity in the Internet era and breaking through the argument that digital technology can enhance individual creativity. Practically, this study also hopes to inspire people who are using this kind of digital technologies.

This study starts with the technologies of online creative idea generation. We advocate that online creative idea generation technologies should be regarded as actors, who have activities just like humans. As a form of media, it disciplines our creative activities and opens up the possibilities of creative activities online. Therefore, we must recognize that online creative products are the result of the co-creation of machines and humans, and think further about our relationship with technology. More importantly, online creative idea generation technologies have been involved in mass psychology and are affecting our thinking patterns. This is because, first, technology imposes a certain restriction on humans. Traditional creative idea generation requires people to have a rich imagination. Online creative idea generation seems to make it possible for everyone to be creative quickly, but at the same time, it limits the possibilities for a wider range of creativity. Second, online creative idea generation technologies influence mass psychology and behavior through material metaphors. The online creative idea generation programs are more like black boxes, in the sense that people know how to use them, but most do not know how it works. This is especially helpful for people who are less creative. These analyses collectively point to an obscure fact that creativity is becoming less and less important in online creative idea generation activities. Therefore, it is crucial to be alert to the degradation of people’s creativity. In addition, one should remember that maintain a prudent attitude and a critical manner to avoid falling into the trap of material metaphors.

This study makes a bold breakthrough and tries to analyze the possibility of studying online creative idea generation from the perspective of materiality, hoping to open up research ideas for scholars. However, the study is limited in several aspects. First, our study is based on the analysis of the concept of media materiality, and there is no empirical data to confirm whether and how people’s creativity declines in online creative idea-generation activities. This can be one of the future research directions. Second, it is hard to provide the most effective way not to be constrained by digital technology and avoid stepping into material metaphors. In future research, more advanced methods and analyses should be considered to dissolve this problem. Despite the limitations, the study contributes to the literature on online creative idea generation theoretically and practically and enriches our understanding of online creative idea generation.

## Data availability statement

The original contributions presented in this study are included in the article/supplementary material, further inquiries can be directed to the corresponding author.

## Author contributions

SL designed the study and wrote the manuscript. FJ and CZ provided intellectual input and participated in the discussion. All authors have read and approved the manuscript.
